# Role of Graphene Topography in the Initial Stages of Pentacene Layer Growth

**DOI:** 10.1021/acsomega.3c03174

**Published:** 2023-07-24

**Authors:** Manisha Chhikara, Gvido Bratina, Egon Pavlica

**Affiliations:** Laboratory of Organic Matter Physics, University of Nova Gorica, Vipavska Cesta 13, SI-5000 Nova Gorica, Slovenia

## Abstract

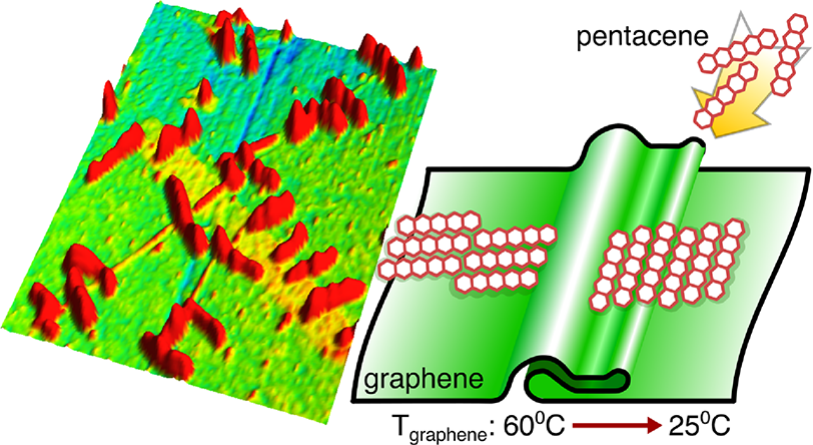

Using atomic force microscopy, we probed the growth of pentacene molecules on graphene that was fabricated by chemical vapor deposition and transferred onto 300 nm-thick SiO_2_ substrates. The topography of such graphene has two important properties. First, its surface is comprised of folds that have different orientations, and second, it has several multilayer-graphene regions distributed over the monolayer-graphene surface. On such folded graphene features, we vacuum evaporated pentacene and observed three-dimensional islands with an average height of ∼15 nm. They are oriented either parallel or perpendicular to the folds, and they are also predominantly oriented along the symmetry axes of graphene. Orientation of pentacene islands on graphene evaporated at room temperature has a wide distribution. On the contrary, most of the pentacene islands evaporated at 60 °C are oriented at 30° with respect to the fold direction. We observed that the folds act as a potential barrier for the surface transport of pentacene molecules. In addition, we interpret the 3D growth of pentacene islands on graphene in terms of reduced polar components of the surface energy on graphene investigated with contact angle measurements.

## I Introduction

The principal drawback of graphene, precluding it from being used in a widespread manner as a staple material in electronic switching devices, is the absence of the electronic energy band gap and negligible density of states at the principal points of the Brillouin zone. An increase in the on/off ratio of graphene-based field-effect transistors could in principle be achieved by integration of organic semiconductor (OS) molecules with graphene.^1,2^ Most often, the organic molecules are attached to the graphene surface by the π–π interaction, which leads to a non-negligible charge transfer at the graphene/OS interface. The density and areal configuration of transferred charges is crucially dependent on the molecular arrangement on the graphene’s surface, i.e., order/disorder is one of the principal parameters characterizing the graphene/OS interface. Therefore, it is crucial to understand the adsorption of organic molecules on graphene. Pentacene is one of the organic molecules that forms well-ordered polycrystalline layers with a relatively high charge carrier mobility on a variety of substrates. This stimulated a considerable interest in pentacene-based organic thin-film transistors.^1−3^ However, the growth of pentacene is highly influenced by the topography of graphene and the substrate underneath.^4−6^

While mechanically exfoliated graphene presents itself as mostly defect-free, the tens-of-micrometer-size flakes preclude its use in a large-scale production setting. Chemical vapor deposition (CVD) instead yields large-scale graphene foils, which are frequently plagued by folds. There are several factors that contribute to the formation of folds. First, differences in thermal expansion coefficients of copper (or nickel) foil used as a template for graphene growth induce strain in graphene layers. Second, folds may also emerge during the transfer process of graphene to the desired substrate and may exhibit chemical reactivity superior to flat, defect-free graphene.^7^ Third, trapped molecules (hydrogen and hydrocarbon) between graphene and copper during the CVD growth process can be another reason for such unusual fold formations on graphene.^8^ The 6-fold symmetry of the graphene unit cell is broken at folds, giving rise to the triangular pattern of atoms, while flat graphene exhibits a honeycomb lattice.^9^ In a triangular pattern, out of six carbon atoms, only three are observed in honeycomb rings. Nirmalraj et al.^10^ have highlighted the influence of folds on the growth of pentacene and reported a large molecular energy gap for pentacene deposited on high folds (1–3 nm thick) as compared to flat graphene. They measured a large conductance gap of pentacene molecules deposited on such folds (3.0 ± 0.3 nm) in comparison to that on flat graphene regions (1.3 ± 0.2 nm). This is due to the increased spatial distance at folds that leads to significant screening from the underlying Cu substrate. When pentacene is deposited at such regions of surface exhibiting increased local curvatures (folds), it may acquire a different growth configuration relative to the flat graphene. Moreover, the growth mode may depend on the orientation of folds itself with respect to the flat regions.

In light of the importance of the initial stages of growth of OSs on graphene on the device performance, we elected to explore the growth dynamics of pentacene at low coverage on graphene. Graphene in our studies was composed of a high areal density of folds. It was mostly of a single-layer type with a sizable fraction of a multiple-layer type. Our experimental results confirm that pentacene islands can be aligned in the direction perpendicular to the fold regions and highly depend on the characteristics of the folds. We show here that folds not only attract pentacene molecules but also determine the in-plane direction of the island nucleation and subsequential growth. We also observe that pentacene growth on folds is not hindered by the poly(methyl methacrylate) (PMMA) residues on graphene in the submonolayer regime. Finally, we examined the cause of the Volmer–Weber growth mode of pentacene layers by extracting the surface energy components (polar and nonpolar) of graphene/SiO_2_ and graphene/sapphire by performing contact-angle measurements.

## II Experimental Section

Commercially available CVD graphene on Cu foil (from Graphenea) was transferred onto 300 nm-thick SiO_2_ layers thermally grown on Si(001) substrates using a wet chemical route.^11,12^ A 300 nm-thick layer of PMMA dissolved in chlorobenzene was drop cast onto graphene at 60 °C. After Cu foil was etched in iron chloride solution, the PMMA/graphene sample was left to dry and transferred onto the SiO_2_ substrate at 90 °C. Prior to transfer, the SiO_2_ substrates were immersed for 10 min in a sequence of baths comprising acetone, isopropanol, and deionized water. They were further cleaned in piranha solution for 10 min. PMMA from graphene was cleaned, as described in ref (12). Pentacene (Sigma-Aldrich, >99.9%) layers were deposited on graphene by vacuum evaporation performed in a high-vacuum chamber at a base pressure of 10^–8^ Torr. A typical deposition rate was 1 nm/min. The amount of deposited pentacene was monitored by an in situ quartz-crystal thickness monitor. The morphology of pentacene islands was examined ex situ by atomic force microscopy (AFM), Veeco CP-II operating in a non-contact mode using Si tips mounted on cantilevers with a resonance frequency of 325 kHz and a spring constant of 40 N/m. The relative humidity level was kept in the range of 25–30% during the AFM measurements. Gwyddion software was used to perform the statistical analysis of the pentacene islands.

Selected samples were examined by contact-angle measurements to assess the role of the substrate on the surface energy of graphene. It was determined by measuring the contact angle of deionized water, diiodomethane, and ethylene glycol. A μm-sized liquid drop was released from a microsyringe onto the substrate surface. Images of a liquid drop were obtained using a charge-coupled device camera. The liquid contact angle was determined by measuring the angle between the tangent line and the droplet–substrate interface line.

## III Results and Discussion

**Figure 1:**
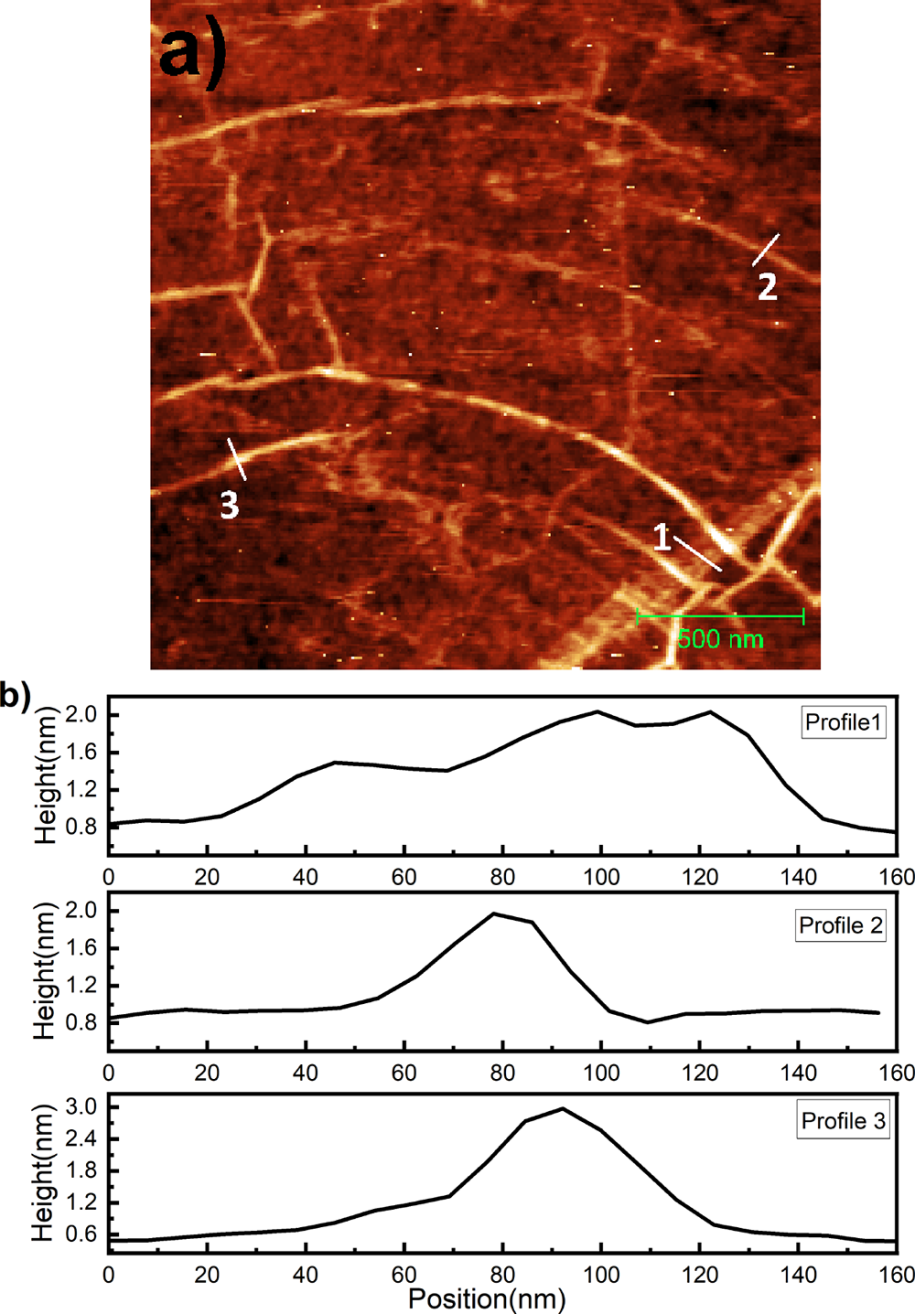
(a) 2 × 2 μm^2^ AFM image of CVD graphene transferred onto 300 nm-thick SiO_2_. The bright features represent the folds in graphene. The height scale is 5 nm. (b) Height profile of the geometry of various folds on graphene of the surface type (panel a) along line profiles 1, 2, and 3, respectively.

Figure 1a shows a representative topography of pristine CVD graphene on 300 nm-thick SiO_2_ recorded by AFM. The scan size is 2 × 2 μm^2^, and the bright tones represent the higher elevations; the height scale (black-to-white) is 20 nm. Apart from atomically smooth regions, we see a network of bright lines of different widths and heights. These features are folds in graphene. The position of such folds is highly influenced by the topography of the underlying Cu substrate that is preserved after transferring to the SiO_2_/Si substrate.^12,13^ Folds have variation in the height and width. The mean height of the folds is in the range between 0.4 and 2.5 nm, and their width was found to vary in the range between 50 and 310 nm. So the fold height ranges from a simple wrinkle (0.4 nm) to a multilayer graphene (MLG) (1.3 nm). Such a variance in morphology is expected and is widely described in previous reports.^11,14,15^ Kim et al.^15^ performed electron diffraction upon scanning an electron beam across the folds. Inside the folds, the intensity of the diffraction spot was twice as compared to the regions outside the fold. For example, in a trilayer fold, they observed a set of three hexagonal diffraction spots, and the total intensity of the diffraction spots was three times larger than that of a single spot diffracted off a flat monolayer. Another set of hexagonal spots appears from the middle layer of the fold. The intensity inside the folded region is twice that of monolayer graphene, and the spot shows splitting of two distinct diffraction spots with a small angle rotation of 0.4°. A simple folded geometry is the most frequent and results in moderately higher and wider features under the AFM tip. If the amount of the folded graphene exceeds a critical value, such folds collapse and form bilayer or even trilayer folds, which appear lower than simple folds but wider. Such a variance in the geometry of the folds is exemplified in Figure 1a. We extracted quantitative information on such folds by measuring the height and width at different positions. Figure 1b represents the height profile of one such wide fold along line 1. Such a fold has a height difference of ∼1.2 nm and a width of ∼130 nm. Such a height difference corresponds to the thickness of trilayer graphene and is consistent with an earlier report.^15^ The interlayer interactions in folds and strain as a result of the bending of the fold play crucial roles in the final shape of the fold. The height profiles 2 and 3 represent the fold along the line profiles 2 and 3, respectively. They are taller and narrower as compared to the fold on line profile 1. Such folds have a range of heights varying from 1 to 2.5 nm and widths from 60 to 90 nm. Such higher and narrower features are intermediate “standing collapsed wrinkle”.^13^ Their height is large enough, so they collapse but remain standing below a certain height of 8 nm based on energy calculations of interlayer interactions and elastic bending.

**Figure 2:**
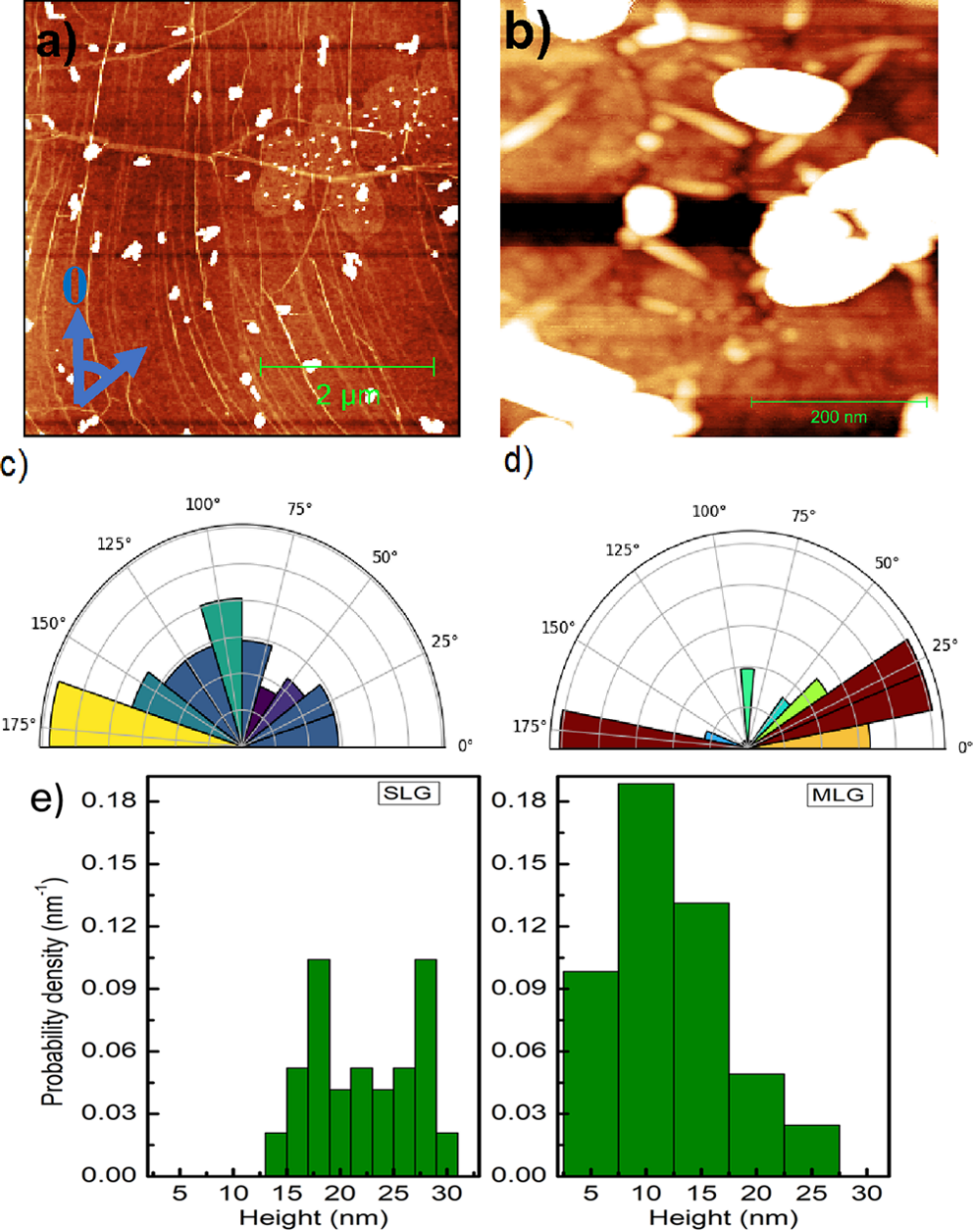
(a) 5 × 5 μm^2^ AFM topography image of submonolayer growth of pentacene on CVD graphene at room temperature. (b) High-resolution 500 × 500 nm^2^ AFM image of pentacene islands on graphene. The height scale is 20 nm. The white features correspond to the pentacene islands. The thin lines on the graphene surface are various kinds of folds that attract pentacene molecules. (c) Polar histogram for the orientation distribution of pentacene on graphene relative to the vertical axis in the AFM image. (d) Polar histogram of the orientation of folds on graphene. (e) Probability density histogram of island height on SLG (leftmost histogram) and MLG (rightmost histogram). The data were obtained from the island shown in panel (a).

Onto the graphene surface exemplified in Figure 1a we have deposited pentacene, taking care to remain in the submonolayer coverage. Figure 2a shows a representative topography obtained by AFM of a surface comprising a 0.03 monolayer (ML) of pentacene. The scan size is 5 × 5 μm^2^. Pentacene was deposited at a substrate temperature of 25 °C. Pentacene forms three-dimensional (3D) islands, which are represented in the image as white irregularly shaped features and are located near or on the folds. From this, we speculate that the onset of island nucleation occurs at or in close proximity of the folds. Islands grow in close proximity to the folds. These islands are of various shapes from circular to elongated rod-like structures. Some of them extend from the top of the fold away from it, while some of them tend to align along the fold lines. Similar results have been confirmed with other molecules also where folds serve as a template for the growth of organic molecules.^16^ Khokhar et al.^17^ reported that folds play a critical role for the growth of 4,4′-bipheneyldicarboxylic acid (BDA) on graphene grown on Ir(111). Essentially, folds represent preferential sites for the nucleation of organic molecules. The local variations in strain on or near the folds in graphene reduce surface mobility of the molecules as compared to defect-free regions.^18^ Therefore, folds effectively immobilize the incoming molecules, thereby increasing the probability for island nucleation and finally leading to a 3D island growth.

In addition to the folds on graphene in Figure1a, we also observe MLG regions on graphene similar to that reported earlier.^11^Figure 2a shows small pentacene islands (white circular features marked with a blue arrow) on such MLG regions. We note that the size of pentacene islands is considerably smaller and their density is considerably higher as compared to that on single-layer graphene (SLG). The island density on SLG is 2.2 μm^–2^, and the mean island area is 0.15 μm^2^. On the other hand, the island density on MLG is 22.5 μm^–2^, and the mean island area is 0.002 μm^2^. This indicates that indeed there is a strong drive for the pentacene molecules to preferentially form islands. Such a 3D island formation corresponds to the Volmer–Weber growth mode on graphene.

Figure 2b represents a 500 × 500 nm^2^ high-resolution AFM image of pentacene islands on smaller folds, which are ubiquitous on graphene. The deposition conditions were kept the same as in Figure 2a. Such smaller folds are less visible in a large-size image. Their height is between 0.5 and 3 nm, and their lateral size is up to 100 nm. We note that pentacene islands start to form on top of these folds and continue to grow in a direction perpendicular to the length of the fold. We can therefore speculate that pentacene islands start to nucleate in the vicinity of the folds. The majority of the islands are ∼30 nm high. Therefore, we can conclude that pentacene islands are formed mostly on the folded features of graphene. Furthermore, we also studied if they have any preferred orientation. The polar histogram in Figure 2c represents the angular distribution of the orientation of the long axis of islands with respect to the vertical axis in the AFM image. The zero angle is marked (blue) on the AFM image (Figure 2a) to connect the image and polar histogram. A significant number of islands are aligned at 172 ± 8° with respect to the vertical axis. However, most of them seem to have random orientation on graphene. So, we can conclude that they do not have any preferred orientation on graphene at room temperature. The polar histogram in Figure 2d represents the orientation of the folds on graphene with respect to the vertical axis in the AFM image. Most of the folds have two preferred orientations: 175 ± 5 and 20 ± 8° relative to the vertical axis. Some of them are oriented at 6 ± 2° relative to the vertical axis. Therefore, the orientation of a majority of the folds is along the crystallographic axis of graphene. If we compare the orientation of islands with respect to the folds from Figure 2c,d, we can find that many islands are aligned along the directions of the folds, while the rest have a wide distribution.

We also compared the height distribution of pentacene islands on SLG and MLG regions, as shown in Figure 2a. The respective histograms for the probability density of island height are shown in Figure 2e. The leftmost histogram shows the data obtained on SLG, and the rightmost histogram shows the data on MLG. A polynomial background was subtracted excluding the marked islands on the AFM image, and the resulting background plane was set to zero. The height of the islands was extracted comparative to that of this background plane. A significant difference in the height distribution of the islands on the SLG and BLG regions is evident. We observed a bimodal distribution of islands on SLG where the leftmost peak at ∼18 nm corresponds to lower height islands with an additional peak at ∼28 nm. The height distribution of the islands on MLG instead exhibits a slightly left-skewed distribution with a maximum at around 10 nm. Islands on MLG (about 10 nm) have a lower height in comparison to SLG (around 20 nm). Such a behavior has been observed before^6^ and was interpreted in terms of the reduced influence of the interface dipole field on the surface energy of graphene, thereby lowering the molecular surface mobility on MLG. This results in smaller islands on this region of graphene.

**Figure 3:**
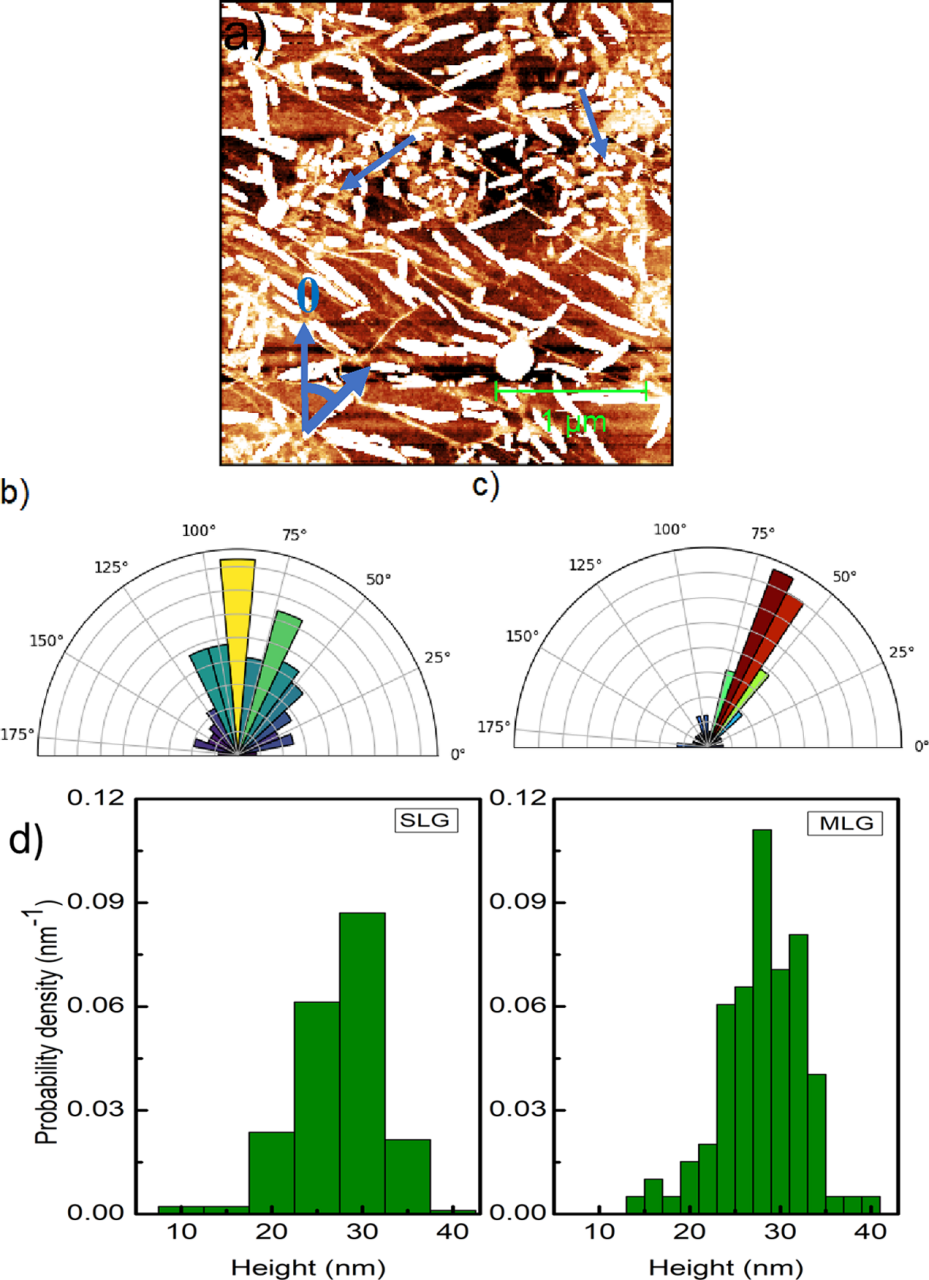
(a) 3 × 3 μm^2^ AFM image of 3D pentacene islands evaporated on CVD graphene at a 60 °C temperature. The height scale is 20 nm. The white rod-like features represent the pentacene islands on folds. (b) Histogram showing the angular distribution of islands on the graphene layer relative to the vertical axis of the image shown in panel (a). (c) Polar histogram of the angular distribution of folds in graphene. (d) Probability density histogram of the island height on SLG (leftmost histogram) and MLG (rightmost histogram). The data were obtained from islands shown in panel (a).

Elevating the temperature during pentacene evaporation has an important effect on all three key parameters affecting the nucleation, growth, and consequently the final morphology of thin layers: molecular surface mobility, their desorption rate, and the molecule–substrate interaction strength. Indeed, as we increased the substrate temperature to 60 °C during evaporation of pentacene, the resulting layer exhibited substantially different morphologies relative to the room-temperature evaporations. This is exemplified in Figure 3a, which shows a 3 × 3 μm^2^ AFM topography map of pentacene layer grown on graphene at substrate temperature *T*_s_ = 60 °C. The surface coverage is 19.5%. We see that the layer morphology is characterized by elongated islands (bright features) aligned in close proximity to the fold lines. The island density is 22.8 ± 1.8 μm^–2^, while the mean island area is 0.008 μm^2^. The mean value of the length of the longer island axis is 84 ± 5 nm, and the average island height is 15 ± 2 nm. The surface mobility of the molecules increases at a higher substrate temperature during growth. On the other hand, a confining effect of the folds is still present and appears to be dominating, causing the molecules to attach to those already immobilized near the folds. This leads to the formation of elongated islands. The interaction of the molecules with graphene contained in folds is so strong that it precludes the lateral growth of the islands, thereby favoring a 3D island formation. Interestingly, this effect is much less evident on the MLG regions (shown by blue arrows in Figure 3a). There, the islands are significantly smaller in size than SLG and do not exhibit any preferential orientation. The island density is 64.6 ± 0.5 μm^–2^. The coverage is 19%. With increasing substrate temperature, the islands are elongated and the mean size is 52 ± 4 nm. Furthermore, we note that they have preferred orientations on graphene. This is evident from their long-axis orientation distribution. The polar histogram plot in Figure 3b represents the distribution of the angle between the long-axis and vertical direction of ∼200 pentacene islands. Hence, 0° and 180° indicated the orientation of islands along the vertical axis in the AFM image, while 90° indicates the orientation of islands along the horizontal axis. We obtained a peak distribution of islands aligned in the horizontal direction of 90 ± 5° (yellow color bar). Additionally, we see a significant distribution of islands in the direction of ∼68 ± 5° (shown in green color bar). The rest of the islands exhibit a broad angle distribution between 40 and 120° (dark green color bars). Only a few islands (less than 10%) are oriented in larger angles. Apparently, their preferred direction is horizontal and ∼25° off. In addition to the island orientation on graphene, we also plotted the polar histogram to determine the alignment of the folds on graphene. The density of folds is 5.8 ± 0.3 μm^2^ on graphene in Figure 3a. Figure 3c represents the histogram for the orientation of folds; we note that most of the folds are elongated in 61 ± 4 and 64 ± 4° directions (red and brown color bars) in the AFM image. And very few are oriented in the vertical direction. If we note the orientation of pentacene islands with respect to the folds in graphene from Figure 3b,c, we can observe that the majority of the pentacene islands are oriented at 30° with respect to the folds. In addition to the alignment of the pentacene islands, we also investigated their height distribution on SLG and MLG as the substrate temperature increased to 60 °C. Figure 3d represents a histogram showing the probability density of the island height on SLG and MLG, leftmost histogram and rightmost histogram, respectively. We see that the height distribution (leftmost) has a peak at around 30 nm and the width of the distribution narrows as compared to the height distribution to that at room temperature. Both distributions exhibit slight right-skewness, indicating that the growth of larger islands is favored. Interestingly, both distributions exhibit peaks at similar heights (approximately 27 nm). This suggests that at 60 °C, the difference between SLG and MLG is the influence of the dipolar electric field that decays as the third power of the distance from the SiO_2_/graphene interface becomes negligible. The heat-induced surface energy becomes nearly equal for both types of graphene. This results in a similar height distribution of pentacene islands in both regions.

**Figure 4:**
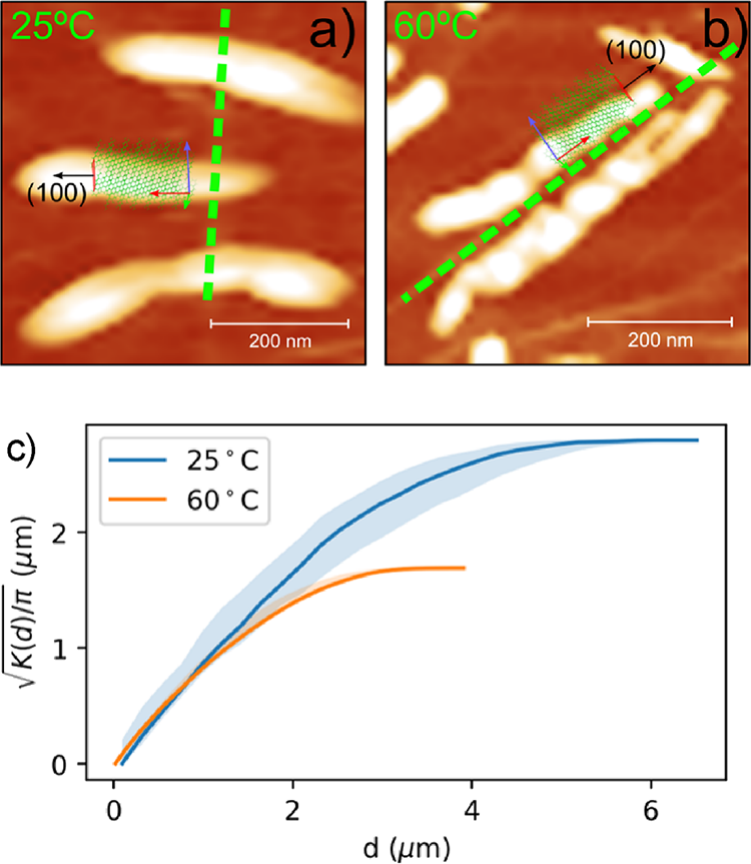
Close view of AFM of representative pentacene islands on graphene evaporated at room temperature (a) and at 60 °C (b). The principal directions of the folds are indicated with dashed green lines. The normal of the (100) lattice plane, which represents the fastest growth direction of pentacene islands, is labeled with a black arrow. The height scales are 20 and 30 nm for panels (a) and (b), respectively. (c) The solid lines represent the square root of Ripley’s K function, divided by π, as a function of the distance between islands evaporated at substrate temperatures of 25 and 60 °C, respectively. The filled area represents a range obtained by a Monte Carlo simulation of randomly distributed islands.

Figure 4 shows an AFM close-up of a few pentacene islands deposited at room temperature (a) and 60 °C (b). The green dashed lines show the direction of folds. Drawings of pentacene unit cells are superimposed with the principal axes of the pentacene unit cell (a, b, c) represented by red, green, and blue arrows, respectively. Similar studies have been performed on pentacene and other organic molecules during its initial stages of growth on graphene using grazing incidence small and wide-angle X-ray scattering.^19−22^ Schematic representation of the pentacene unit cells in these highly anisotropic islands is based on the findings of ref (19). In the absence of our high-resolution data, we rely on the findings of ref (19) and can conclude that the normal of (100) facets represents the direction of a rapid growth and the pentacene unit cell is oriented in pentacene islands, as shown in Figure 4. We can speculate about only the alignment of pentacene molecules in islands. Presumably, the pentacene molecules align, as schematically presented in Figure 4a,b. The distances between pentacene islands were analyzed in terms of Ripley’s K function.^23^ The analysis demonstrates that the distribution of distances between pentacene islands (solid line in Figure 4c) falls within boundaries (shaded area in Figure 4c), calculated by a Monte Carlo simulation under the assumption of randomly distributed islands. Hence, we conclude that pentacene islands are evenly distributed on top of graphene. The island density is 1.9 ± 0.3 μm^–2^ at 25 °C and raises to 22 ± 2 μm^–2^ at 60 °C. More importantly, the rise of the temperature does not alter the positions of nucleation sites, which remain uniformly distributed.

**Figure 5:**
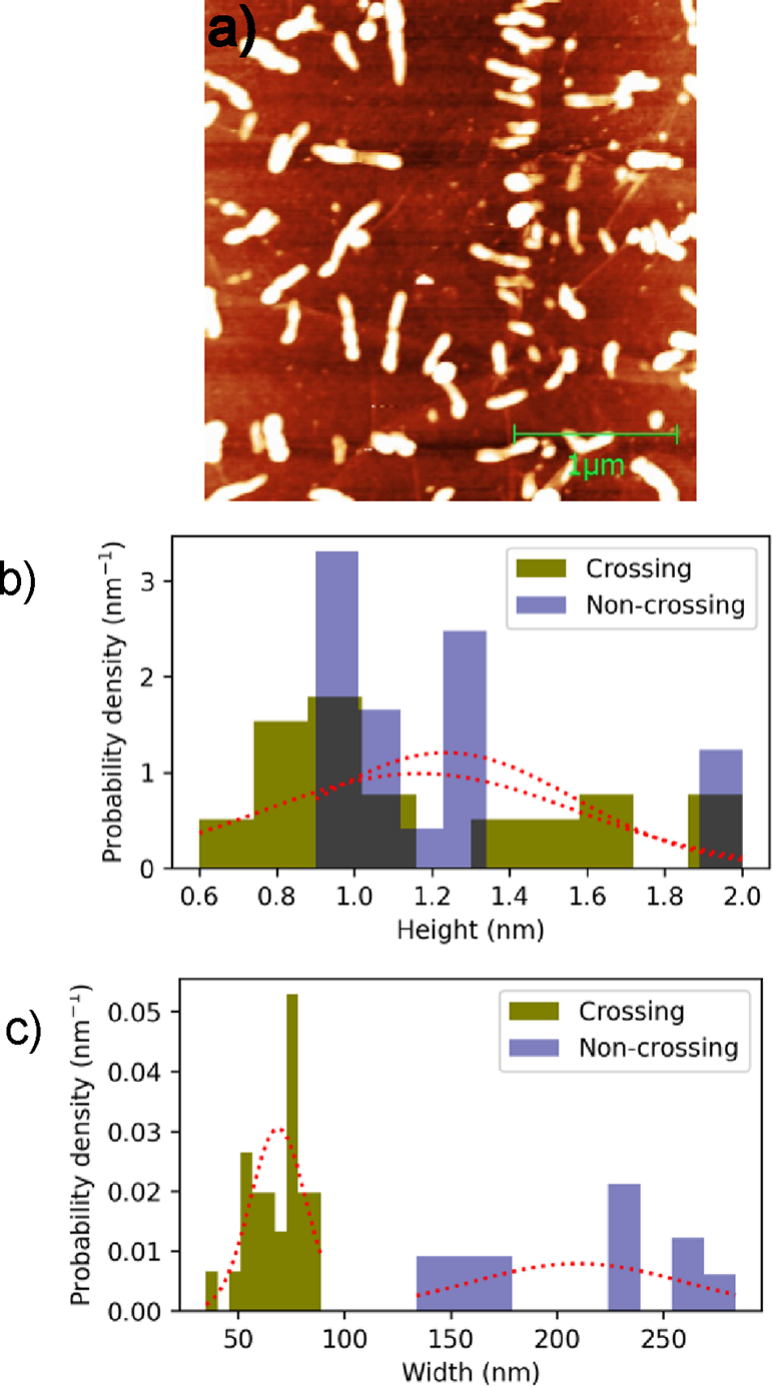
(a) 3 × 3 μm^2^ AFM image of 3D pentacene islands evaporated on CVD graphene at room temperature. The height scale is 20 nm. The white rod-like features represent the pentacene islands on folds. The average height of islands is ∼15 nm. Clearly seen from the image, pentacene islands have strong tendency to grow perpendicular to folds. (b) Distribution of crossing and noncrossing pentacene islands as a function of the fold’s height. The dashed lines represent a Gaussian function with the corresponding mean and standard deviation. (c) Distribution of crossing and noncrossing pentacene islands as a function of the fold’s width.

In addition to the alignment of pentacene islands along the fold lines, the growth of pentacene islands can occur perpendicular to the fold lines. The AFM micrograph (Figure 5a) displays such an example of the morphology of pentacene islands that are aligned perpendicular to the folds. The mean area of islands is 0.3 ± 0.1 μm^–2^ with an average height of 18.6 ± 4.0 nm. The island density is 6.2 ± 0.5 μm^–2^. Such an alignment of pentacene islands can be observed on both wider and narrower folds. However, we observe that pentacene molecules are unable to cross over wider folds. They have a perpendicular orientation with respect to the fold direction and start to nucleate from the side of the fold that is higher. However, they easily crossover narrower folds. The intersection of the folds is another major source of attraction for pentacene molecules. Several pentacene islands can be seen grown in close proximity to each other at such an intersection of multiple folds. The corresponding alignment on folds can be attributed to the fact that folds might have electronic configurations different from those of the flat graphene. Therefore, the observed pentacene molecular assembly may be guided by the orientation of the folds.

Common to both island orientations, perpendicular to the fold and along the fold is that the islands do not cross the fold unless they are narrow enough. In other words, the interaction of pentacene molecules with graphene on the fold is negligible. To investigate the effect of fold height and width on the ability of pentacene molecules to cross the fold, we have analyzed the distribution of crossing pentacene islands and compared the distribution to noncrossing islands. The results are presented in Figure 5b,c. The dashed lines represent a Gaussian function with the corresponding mean and standard deviation as a guide to the eye. We note that the fold’s height does not affect the ability of pentacene molecules to cross the fold. In fact, the two distributions in Figure 5b are comparable. In contrast, the width of the fold clearly influences the ability of pentacene molecules to cross the fold. The corresponding distributions in Figure 5c are clearly distinct from each other. The mean values of the fold’s width of noncrossing and crossing islands are 210 ± 51 and 69 ± 13 nm, respectively. The difference between the two is more than two standard deviations. To understand this phenomenon, we refer to the literature sources, which considered thermal,^24^ electronic,^14,25,26^ and structural^9,15^ properties of the folds on CVD graphene in more detail. At the outset, we note that folds may present themselves in a variety of forms.^15,25^ When the two graphene domains interact during graphene growth on a metal substrate, a wrinkle may form. If the domain interaction is more intense, one domain may overlap the other,^25^ forming a BLG or even triple-layer graphene.^15^ The folds differ considerably from the graphene flatland in parameters that crucially affect the interaction of adsorbed molecules with graphene and lead to island nucleation. Mechanically, they seem to block the propagation of stress in a folded graphene under the tensile strain.^27^ A detailed investigation of the electronic properties of folds was investigated by Vasić et al.^26^ using Kelvin probe force microscopy. Their results show that the folds act as potential barriers that localize charge carriers within a graphene domain. They observe a significantly increased contact potential difference between the fold and graphene flatland, which could arise from an increased charge carrier concentration within the fold or form an increased-curvature-induced strain on the fold. Chen et al.^24^ investigated the thermal conductivity of suspended CVD graphene membranes by micro-Raman mapping. A selected number of samples comprised folds of unspecified dimensions. Their results show a ∼27% higher thermal conductivity of the fold-free samples relative to the folded samples. This suggests that folds increase the thermal resistivity acting as “hot spots”. This is important, especially when the substrate is heated during pentacene growth. An increased surface energy reduces the sticking coefficient of pentacene molecules and prevents island nucleation.

**Figure 6:**
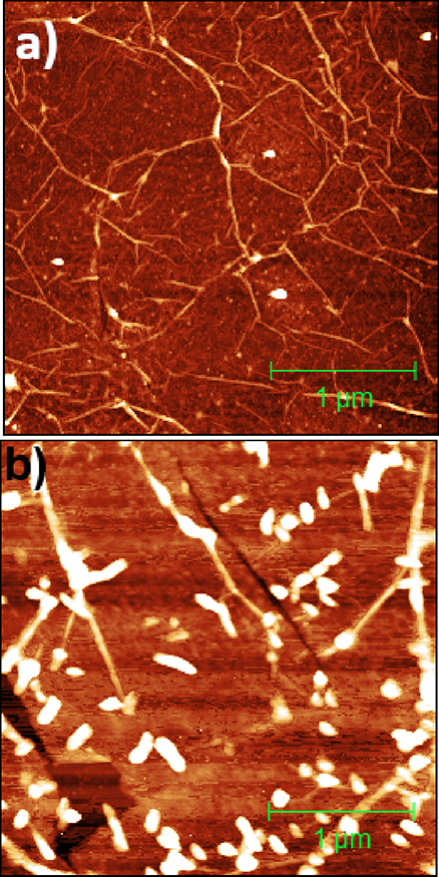
(a) 3 × 3 μm^2^ AFM image of CVD graphene transferred onto the sapphire substrate. The bright ovals are the PMMA residues. The folded curvatures are the high-density ridges. The height scale is 10 nm. (b) 3 × 3 μm^2^ AFM image of pentacene islands on CVD graphene transferred onto sapphire. The height of pentacene islands is 11 ± 2 nm. Pentacene grows in a 3D growth mode on graphene/sapphire. The height scale is 20 nm.

To study the effect of the substrate underneath graphene on the growth of pentacene, we have transferred CVD graphene to sapphire and deposited pentacene at room temperature, as done for Figure 5a. Figure 6a shows a 3 × 3 μm^2^ AFM topography map of pristine graphene transferred onto the sapphire substrate. We observe folds similar to those observed for graphene transferred onto 300 nm-thick SiO_2_ wafers. However, their areal density is much higher in the case of the sapphire substrate. Such a high density of folds was found also on graphene grown directly onto sapphire by the CVD method.^28^ Sapphire is an ultraflat substrate; hence, graphene is closely following its surface. Some folds are a result of different thermal expansion coefficients of graphene and sapphire, while others may have formed due to the wet chemical transfer process onto sapphire, as described earlier. The height of such folds varies from 1 to 12 nm. We have deposited pentacene on such a graphene sample at room temperature. The samples were examined by AFM immediately upon completion of the evaporation. By examining the pentacene layers after 2 weeks, we can confirm that they did not exhibit any morphological changes.

Figure 6b shows a 3 × 3 μm^2^ AFM image of pentacene islands on graphene/sapphire. The density of islands is 137 μm^–2^, and the size of pentacene islands is 78 ± 4 nm. The average height of most of the islands is 11 ± 2 nm. From the morphology, we can extract two important conclusions. First, it is further confirmed that pentacene molecules preferentially nucleate on/near the folds. Second, the islands grow in a 3D growth mode on folds, regardless of the type of the underlying substrate. However, they are smaller in size as compared to that on graphene/SiO_2_ under similar growth conditions. It was reported earlier that the adhesion energy at the interface between sapphire and graphene synthesized by CVD is relatively high (1.47 J/m^2^).^29^ Such high values cannot be reconciled by dispersion forces. It is almost three times higher than that at the interface of SiO_2_ and graphene (0.45 J/m^2^).^30^ We consider that our samples of graphene/sapphire have a slightly higher interfacial energy compared to that on graphene/SiO_2_.

So, far we have analyzed the role of folds on the pentacene morphology. Folds dictate the arrangements of these 3D islands on graphene. Furthermore, we can relate the 3D formation of islands to the surface energy of the substrate. The shape and size of islands may vary depending on the surface energy of different substrates. Rebernik Ribic et al.^31^ have examined the morphology of submonolayer thick pentacene films on polymeric substrates with a lower polar contribution to the surface energy, and pentacene islands appeared more compact and exhibited a strong tendency toward the 3D growth. On strongly polar substrates such as SiO_2_, pentacene nucleates in a layer-by-layer mode, forming strongly dendritic islands.^31−33^

We have, therefore, examined the graphene surface from the standpoint of polar contribution to the surface energy and employed the Owens–Wendt approach.^34^ This approach considers that the free surface energy can be expressed as a sum of polar and dispersive surface energy. The polar component of the surface energy results from the electrostatic intermolecular forces between permanent dipoles and multipoles of molecules and surfaces. In contrast, the dispersive energy is determined by the electrostatic interaction between fluctuating induced dipoles and multipoles. By measuring the contact angle of deionized water, diiodomethane, and ethylene glycol and using the values for the polar and the dispersive energy of the liquids from refs (34) and (35), we have obtained the values for polar and dispersive energies for SiO_2_, graphene/SiO_2_, sapphire, and graphene/sapphire. CVD graphene was used for the contact angle measurement as it can be transferred in a large area on a substrate. The results are listed in Table 1. The total surface energy of substrates is 66 mJ/m^2^ for SiO_2_ and 42 mJ/m^2^ for graphene/SiO_2_. The magnitude of the polar component of the two substrates differs considerably and was found to be 43.53 ± 6.90 and 3.64 ± 1.70 mJ/m^2^ for SiO_2_ and graphene, respectively. We have also measured the surface energy of same liquids on sapphire and graphene transferred onto sapphire. The magnitude of the polar component of surface energy on sapphire and Gr/sapphire also differs (29.11 ± 8.60 and 2.83 ± 2.00 mJ/m^2^, respectively). We can conclude the following: (1) Graphene is nonpolar and the pentacene–graphene interaction is lower on a nonpolar surface in comparison to a polar surface. Graphene transferred onto SiO_2_ and sapphire has a similar but very low polar component of surface energy. The 3D island formation is a result of the weak molecule–substrate interaction. (2) Due to the lower surface energy of graphene on sapphire (36.77 ± 6.40 mJ/m^2^) as compared to graphene on SiO_2_ (42.38 ± 8.00 mJ/m^2^), we observe smaller islands on the former surface; as a consequence of the reduced surface energy, the surface diffusivity of pentacene molecules is lower on graphene on sapphire than on graphene on SiO_2_.

**Table 1:** Values for Polar, Dispersive, and Total Surface Energies for SiO_2_, Sapphire, and Graphene (Gr) Obtained from Measurements of the Contact Angle of Deionized Water, Diiodomethane, and Ethylene Glycol and Using the Values for Polar and Dispersive Energies from Ref (34)^a^

substrate	polar component of surface energy(mJ/m^2^)	dispersive component of surface energy(mJ/m^2^)	total surface energy(mJ/m^2^)
SiO_2_	43.53 ± 6.90	22.85 ± 7.50	66.39 ± 15.40
Gr/SiO_2_	3.64 ± 1.70	38.74 ± 6.30	42.38 ± 8.00
sapphire	29.11 ± 8.60	17.47 ± 7.30	46.58 ± 11.30
Gr/sapphire	2.83 ± 2.00	33.94 ± 6.00	36.77 ± 6.40

a The errors quoted arise from the fitting procedure to the linear function.

## Conclusions

Our systematic AFM study of the initial stages of the growth of pentacene on CVD graphene reveals the 3D growth mode on the folded regions of graphene and on the MLG patches. Pentacene islands can grow either parallel or perpendicular to the folds. At room temperature, most of the islands have no preferred orientation. But evaporating at 60 °C, the majority of them are aligned with 90 ± 5°, i.e., horizontally in the AFM image. In addition, we observe that the growth mode is also not influenced by the change of the substrate after CVD graphene was transferred to sapphire. We obtained a similar 3D growth on sapphire substrates confining most of the islands on folds. We interpret the nucleation of pentacene on folds as a result of the reduced mobility of pentacene due to the local strain present at the folds. Also, with contact angle measurements on graphene, a low polar contribution (3.64 ± 1.70 and 2.83 ± 2.00 mJ/m^2^) of surface energy could be a possible cause of the 3D growth of islands. The lower surface energy of graphene/sapphire (36.77 mJ/m^2^) results in smaller islands as compared to those on graphene/SiO_2_ (42.38 mJ/m^2^).
